# Affective Aspects of Perceived Loss of Control and Potential Implications for Brain-Computer Interfaces

**DOI:** 10.3389/fnhum.2017.00370

**Published:** 2017-07-19

**Authors:** Sebastian Grissmann, Thorsten O. Zander, Josef Faller, Jonas Brönstrup, Augustin Kelava, Klaus Gramann, Peter Gerjets

**Affiliations:** ^1^LEAD Graduate School and Research Network, University of Tübingen Tübingen, Germany; ^2^Team PhyPA, Biological Psychology and Neuroergonomics, Berlin Institute of Technology Berlin, Germany; ^3^Laboratory for Intelligent Imaging and Neural Computing, Columbia University New York, NY, United States; ^4^Hector Research Institute of Education Sciences and Psychology, Faculty of Economics and Social Sciences, University of Tübingen Tübingen, Germany; ^5^Biological Psychology and Neuroergonomics, Berlin Institute of Technology Berlin, Germany; ^6^Center for Advanced Neurological Engineering, University of California, San Diego La Jolla, CA, United States; ^7^Leibniz-Institut für Wissensmedien, University of Tübingen Tübingen, Germany

**Keywords:** brain-computer interface (BCI), electroencephalography (EEG), loss of control (LOC), frontal alpha asymmetry (FAA), independent component analysis (ICA)

## Abstract

Most brain-computer interfaces (BCIs) focus on detecting single aspects of user states (e.g., motor imagery) in the electroencephalogram (EEG) in order to use these aspects as control input for external systems. This communication can be effective, but unaccounted mental processes can interfere with signals used for classification and thereby introduce changes in the signal properties which could potentially impede BCI classification performance. To improve BCI performance, we propose deploying an approach that potentially allows to describe different mental states that could influence BCI performance. To test this approach, we analyzed neural signatures of potential affective states in data collected in a paradigm where the complex user state of perceived loss of control (LOC) was induced. In this article, source localization methods were used to identify brain dynamics with source located outside but affecting the signal of interest originating from the primary motor areas, pointing to interfering processes in the brain during natural human-machine interaction. In particular, we found affective correlates which were related to perceived LOC. We conclude that additional context information about the ongoing user state might help to improve the applicability of BCIs to real-world scenarios.

## Introduction

Traditionally, brain-computer interfaces (BCIs) have been developed to translate measured brain activities into commands for technical systems in real-time. By using measures of neural activity like the electroencephalogram (EEG), BCIs therefore offer alternative communication channels for human-machine interaction (Wolpaw et al., 2002). The primary goal in developing BCI systems was to support persons with severe behavioral impairments like amyotrophic lateral sclerosis (also known as Lou Gehrig’s disease). However, recently there have been developments toward systems that can benefit users without disabilities as well.

From a user perspective, there are three main types of BCIs (Zander and Kothe, 2011): first, reactive BCIs, tracking users’ attention towards stimuli that are externally presented and encode direct control commands. Second, active BCIs relying on voluntarily induced changes in brain activity, e.g., by imagining motor activities. Both active and reactive BCIs are used to control devices through directed commands (e.g., P300 speller; Belitski et al., 2011, or motor imagery based BCIs; Pfurtscheller and Neuper, 2006). Third, passive BCIs apply mental state monitoring to establish an implicit control loop. Passive BCIs do not rely on a user‘s awareness about the concrete information exchanged during this interaction. An example can be found in Zander et al. (2016), where ERP-based error detection was used to improve cursor movements toward targets in a 2d-grid. The participants of the study did not know, that they provided information to the system, but still recognized that system performance was improved. Other possible application scenarios for passive BCIs are adaptive learning environments (Gerjets et al., 2014), automatic correction of errors (Parra et al., 2003) or even the detection of mental states like bluffing in a game context (Reissland and Zander, 2009).

### The Relevance of Complex Mental User States to BCI Performance: The Case of Loss of Control

Most available BCIs, whether reactive, active or passive, try to identify specific, narrow mental states or processes, as for example motor imagery (Lotte et al., 2010), working-memory load (Gerjets et al., 2014) or affective responses (Mühl et al., 2014). However, such systems typically do not account for contextual fluctuations in other mental states and processes that arise during use. In line with this reasoning, previous work suggests that spontaneously occurring decrements in BCI reliability could be related to modulations in unaccounted mental processes and states (Zander and Jatzev, 2012; Myrden and Chau, 2015).

Natural environments for innovative BCI applications (e.g., classroom or workplace settings; see Gerjets et al., 2014) usually contain various factors that can potentially evoke complex mental states in a user. Therefore, such application scenarios might easily induce a variability of different mental states that are not directly related to the core mental state or process that is in the focus of a certain BCI application. Accordingly, such factors can produce changes in EEG signals that are picked up by a system, but cannot be interpreted correctly due to the limited user state model (usually consisting of a single mental state or process) the system is built upon.

As many available classification algorithms assume stationary signal properties, which are learned from a single calibration session, these algorithms are unable to track shifts in the feature space (called non-stationarities) and rather require users to adapt to unexpected or erroneous behavior of the system, for instance by trying to produce brain signals similar to those used during calibration (Krauledat et al., 2006).

Future systems might be able to automatically adapt to contextual changes in the EEG and provide stable performance even in noisy environments outside the lab. To facilitate the development of such systems it seems necessary to investigate complex user states with the aim to separate contextual and potentially interfering states from the primary interaction mode.

In this article, we address the role of one interfering mental state on BCI performance in a paradigm that was designed to experimentally manipulate an important complex user state in the context of BCI systems, namely the perceived feeling of a loss of control (LOC). The LOC paradigm used in our study has already been hypothesized to evoke affective as well as cognitive responses (Zander and Jatzev, 2009) and thereby appears to be particularly suited to study different basic components of complex user states. In this paradigm, the feeling of LOC is evoked by means of a feedback manipulation: subjects were first trained to use a fixed set of rules (color-angle associations) to control a simple letter rotation task by means of button presses with the left vs. right hand. Later in the experiment, the previously learned rules were temporarily violated, thus eliciting the feeling of LOC. This paradigm allows to study the neural signatures of motor executions with and without LOC in order to analyze what happens to a specific BCI signal of interest (in this case motor execution responses) when other cognitive or affective mental states overlap with this signal.

Contrary to standard BCI approaches, we decided to analyze motor execution responses rather than motor imagery responses in order to better control for the perceived LOC. Input based on motor imagery can be rather unreliable, whereas button presses are an extremely accurate input modality thereby not yielding unintended LOC experiences. Moreover, earlier work by Pfurtscheller and Neuper (1997) has demonstrated that that motor execution responses are structurally similar to motor imagery responses with respect to their neural signature. Therefore, for the purpose of the current study, namely to investigate the role of interfering mental states on the stability of BCI performance, we assumed that using a button-press paradigm would yield similar results than using a paradigm based solely on motor imagery.

In earlier work it has already been demonstrated that EEG signature of motor execution responses changed under LOC, thereby leading to a BCI performance degradation when the interface was controlled by EEG signals (Zander and Jatzev, 2012). However, in this work it remained unclear, which aspects of the complex user state contributed to the altered signature of motor responses in the EEG signal.

Following previous work by Zander and Jatzev (2009), we assume in this article that LOC might involve affective processes. According to this reasoning, the feeling of losing control over an interface should provoke negative emotions like irritation, worry, frustration, anger or helplessness (Reuderink et al., 2009). The aim of the current article is to provide additional evidence for this assumption by comparing LOC trials to correct trials in our experimental paradigm with regard to potential neural signatures of affective user states. For this purpose we will not only analyze sensor-based EEG measures but also conduct an EEG-based source analysis.

### Neural Signatures of Potential Affective User States in the Loss of Control Paradigm

Several studies used EEG measures to investigate the neural basis of affective processes (Davidson, 1992; Olofsson et al., 2008; Kim et al., 2013). Laterality in the alpha band measured in frontal regions, also called Frontal alpha asymmetry (FAA), is widely used in these studies as a measure for affective processing. However, this measure seems to capture a wide variety of affective responses. Many studies that used brain lateralization as a measure of affective states or responses focused on one particular dimension of emotional experience, namely the valence dimension (Ahern and Schwartz, 1985; Davidson and Tomarken, 1989; Lin et al., 2008; Huang et al., 2012; Ramirez and Vamvakousis, 2012). The valence (or pleasure) dimension reflects the pleasantness or unpleasantness of an affective event and ranges from extreme pain (or unhappiness) to ecstasy (or extreme happiness). Left frontal activity in this context often indicates a tendency toward positive emotions like happiness (e.g., Ahern and Schwartz, 1985).

However, the equivalence of left frontal activity with positive emotions is not unequivocal. Several studies showed that some emotions with negative valence might also activate the left hemisphere. An interpretation of this apparent contradiction has been provided by the Approach-Withdrawal model, first proposed by Schneirla (Schneirla, 1959), as cited in Dalgleish, 2004). It states that the left hemisphere is active in emotions associated with pleasant or unpleasant approach behaviors like anger or engagement whereas the right hemisphere is related to avoidance behaviors like fear. As an alternative explanation it has been proposed that FAA can be used to measure emotional responses along the dominance dimension (Demaree et al., 2005). Dominance has been specified as “feelings of control and influence over everyday situations, events and relationships vs. feelings of being controlled and influenced by circumstances and others” (Mehrabian, 1994). In this context left-frontal activation is related to more dominant emotions that can also be of positive and negative valence. Whichever of these interpretations is correct, FAA should in any case be sensitive to affective responses we expect to be modulated during perceived LOC. Interestingly, a recent study by Reuderink et al. (2013) found that activity in the lower alpha band was related to the valence dimension, while activity in the upper alpha band was related to the dominance dimension, which might be of important in this analysis, since it seems plausible that LOC might have an effect on the dominance as well as the valence dimension.

Thus, in a first step to explore whether the complex LOC user state contains affective components besides motor activities, we focused on examining a potential FAA shifts occurring during phases of reduced control. Specifically, using the FAA measure, we investigated each individual experience of LOC during phases with LOC (as compared to trials within these phases that do not reflect a LOC).

In a second step, to further substantiate potential affective correlates found using the FAA, we conducted an exploratory cluster analysis on the EEG data to explore whether the corresponding brain dynamics can be attributed to specific neuroanatomical structures. Furthermore, we also wanted to investigate if such brain structures project activity onto areas used to infer the motor execution response in Zander and Jatzev (2012). In order to provide evidence for specific structures as sources of a FAA, we used equivalent dipole modeling of independent components (ICs) derived from an independent component analysis (ICA; Makeig et al., 1996; Oostenveld and Oostendorp, 2002). However, due to the novelty of the experimental paradigm used, it is difficult to make concise predictions about specific brain areas involved, especially since the previous literature on brain laterality concluded that most theories underlying frontal asymmetries lack anatomical specifity (Wager et al., 2003). Nonetheless, it is expected that potentially revealed brain sources underlying an FAA response can be interpreted in the context of the paradigm used in this study.

## Materials and Methods

### Experimental Task

**Figure 1:**
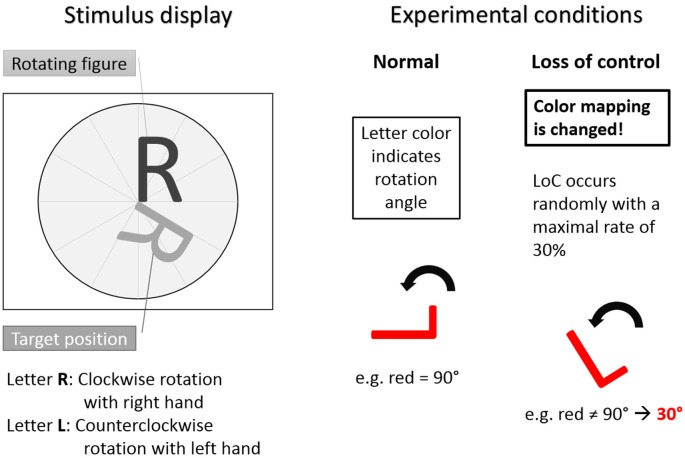
The Rotation-Left-Right game used as experimental task. Left: stimulus display showing stimulus to be rotated and target position. Letter R indicated clockwise rotation with right hand button press. Letter L indicated counterclockwise rotation with left hand button press. Right: experimental conditions. In the normal trials condition the color-rotation angle mapping was fixed. In the loss of control (LOC) trials condition was distorted with a maximal occurrence rate of 30%. Hence, during LOC the subjects did not know how the system responded to a button press. Adapted from Zander and Kothe (2011).

A simple game (called “Rotation-Left-Right”) was used to modulate LOC over the course of the experiment. Subjects had to discretely rotate a letter stimulus until it was aligned to a target letter with regard to its rotation angle. The basic principles of the task are illustrated in Figure 1 and a full description of the experimental task can be found in Zander and Jatzev (2012).

**Figure 2:**
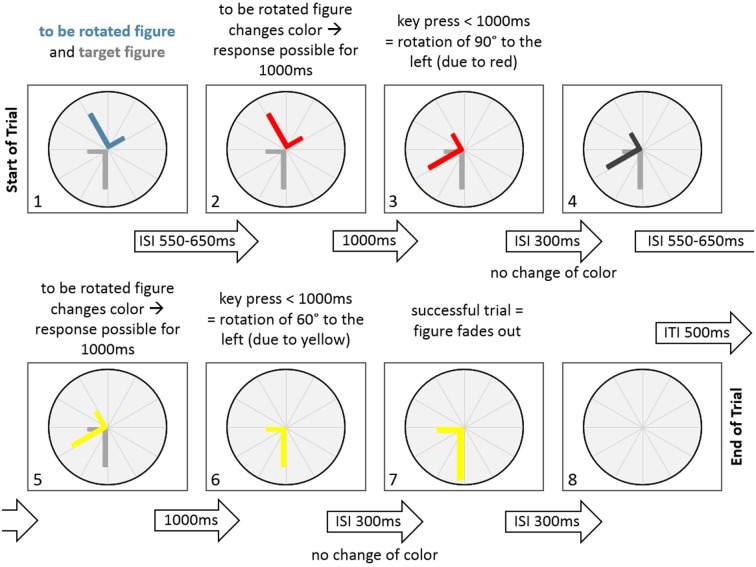
A sample trial of the Rotation-Left-Right game in the normal condition. At the start of the trial the to be rotated figure and the target figure are displayed. Following an inter-stimulus-interval (ISI) of 550–650 ms the to be rotated figure changes color (to red), indicating a potential figure rotation of 90° to the left. After a key press that occurred within 1000 ms, the figure rotates 90° to the left. This is followed by an ISI of 300 ms, where the figure does not change color. After that the figure changes to dark gray in another ISI of 550–650 ms. This is followed by another change of figure color (to yellow), indicating a potential rotation of 60° to the left. Since a key press occurred within 1000 ms, the figure rotates 60° to the left, thereby aligning with the target figure. There is no change in color in the next ISI with a duration of 300 ms. After that the fading out of the figure indicates a successfull trial for 300 ms. Finally, the figure disappears, indicating an inter-trial-interval of 500 ms.

The stimuli were the two capital letters R and L. The capital letter R indicated clockwise rotations after a button press of the right hand and the capital letter L indicated counterclockwise rotations after a button press of the left hand. The color of the rotating letter changed approximately each second to indicate an expected rotation angle with the color being either, green for 30°, yellow for 60° or red for 90°. For each letter rotation, subjects could decide whether they wanted to rotate the rotating letter according to the angle indicated by the color by pressing a button or not. Each trial started with the presentation of a rotating letter (L or R, either colored in green, yellow or red) together with a target position in gray to which the stimulus letter had to be aligned to by means of an appropriate rotation. Subsequently, subjects had a time window of 1000 ms to react (or not) with a left hand (for the letter L) or right hand (for the letter R) button press, resulting in a letter rotation (or not). After 300 ms of the subsequent 850–950 ms interstimulus interval the color of the rotating letter turned to gray. Following the interstimulus interval, a new letter was presented in color to start the next rotation trial. A sample trial is shown in Figure 2.

A training phase was provided to allow subjects to acquaint themselves with the task. All colors and corresponding rotation angles occurred with the same probability and each letter had to be rotated for at least 90° and at most 270° in order to be aligned with a target position. The subjects were instructed to finish the trials as fast as possible. If a subject chose a rotation angle that rotated the figure beyond the target position, then the subject was required to go around the circle another time. In the first part of the experiment, the contingencies between letters, colors and rotations were trained for approximately 30 min. In the second part of the experiment the previously learned rule system of color-angle mapping was systematically violated by rotating the letter by a different than expected angle with a chance of up to 30%. These violations resulted in trials with unexpected rotations (LOC trials), forced subjects to reconsider and potentially modify their previously formed strategies and induced—according to our hypothesis—a complex user state, namely the feeling of a perceived LOC.

Our analysis was focused on data from the second part of the experiment where trials with expected stimulus rotation (normal trials) and trials with erroneous stimulus rotation (LOC trials) were mixed. At the beginning of the analyzed data segment with an overall duration of 15 min, the incidence of trials where previously learned rules were violated was linearly increased from 0% to 30% over 4 min. This was done to prevent disengagement from the task due to an abrupt onset of LOC. Then the occurrence of LOC trials stayed at a maximum of 30% for 7 min, before the incidence of trials with incorrect rotation (LOC trials) was linearly decreased over the next 4 min. On average there were 255.7 (SD = 19.7) trials in the normal condition and 81.8 (SD = 9.4) trials in the LOC condition. There was no statistical difference between left (mean = 121.4; SD = 19.8) and right (mean = 133.2; SD = 19.4) trials in the normal condition. There was also no significant difference between left (mean = 38.6; SD = 9.0) and right (mean = 43.2; SD = 11.1) trials in the LOC condition. Each trial started with a new target position. The average steps to complete a trial were computed for both conditions and it was found that trials in the LOC condition (mean = 4.42; SD = 0.19) had significantly more (*p* < 0.001) steps than trials in the normal condition (mean = 3.99; SD = 0.25). However, there was only a trend towards significance in trial duration (*p* = 0.055) for LOC trials (mean = 10.99 s; SD = 1.26) and normal trials (mean = 10.15 s; SD = 1.19).

### Experimental Setup

#### The Dataset

The dataset comprised 18 healthy subjects (age range: 19–40 years) that were recorded in a previous experiment to investigate the effect of perceived LOC on EEG features related to motor response (Jatzev et al., 2008). The recordings have been approved by the ethical committee of the Berlin Technical University and informed consent has been acquired. Out of these 18 subjects, three subjects had to be removed from the analysis due to strong artifact contamination. EEG-data was recorded with 32 active Ag/Cl-electrodes (positioned according to the extended 10% system) and a biosignal amplifier (Brainproducts GmbH., Gilching, Germany). All channels were referenced to the nasion. Impedances were kept below 20 kΩ. The data were sampled at 1 kHz. The EEG recording sessions lasted for about 66 min, but only 15 min of data that included LOC trials were analyzed for this manuscript.

#### Preprocessing

First, the data was bandpass filtered between 1 Hz and 30 Hz. Then it was down-sampled to 250 Hz and re-referenced to the common average to suppress artifactual activity that spread over all channels (e.g., muscle activity or outside electrical noise; see McFarland et al., 1997). To ensure that our findings could be applied to an online application scenario no further manual artifact rejection was performed. A logarithmic transformation was applied before computing the spectra to better fit the data to a Gaussian distribution. Based on Allen et al. (2004) and the available data, the electrode pairs F3/F4 and FC1/FC2, respectively, were analyzed with regard to potential FAAs. All data processing was done via (MATLAB and Statistics Toolbox Release, 2013; The MathWorks, Inc., Natick, MA, USA) and EEGLAB (Delorme and Makeig, 2004).

### Analysis

**Figure 3:**
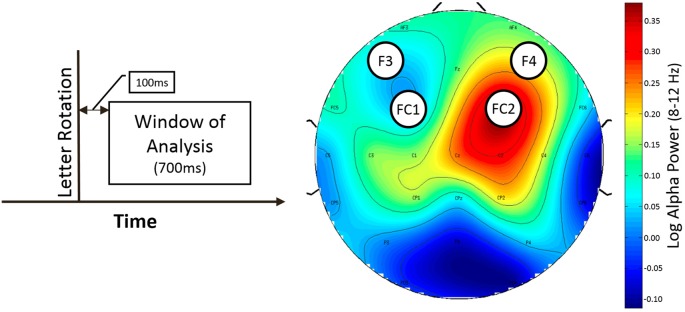
Window of analysis (left) and topografic difference plot illustrating the difference of alpha power between normal and LOC trials (right). Left: time is on the horizontal axis. Vertical line indicates onset of letter rotation. Right: topographic plot showing the found asymmetry effect for trial type as difference in frontal alpha (8–12 Hz) power. Nose is at the top. Channel labels indicate channel location. Electrodes of interest are marked with black circles. Colorbar indicates value range of difference (normal vs. LOC) in alpha power (after logarithm). Blue color shows low alpha power (top left and bottom left). Red color shows high alpha power.

Only rotation trials with button press were used for analysis. The time window for analysis started 100 ms after the rotation of the stimulus and ended 700 ms later (50–150 ms before the interstimulus interval ended) to ensure that the data was not contaminated by effects related to expectancy of the next rotation opportunity (see Figure 3, left).

Power spectral density (PSD) of the EEG signal using Welch’s PSD estimate (Welch, 1967) was used as basis for FAA estimation between 8 Hz and 10 Hz for the lower alpha band and between 10 Hz and 12 Hz for the upper alpha band. FAAs were computed as difference score (ln (right alpha power)) − (ln (left alpha power)), following the approach from Allen et al. (2004). A Kolmogorov-Smirnov-test was used to test the assumption of normality and found that it was not violated.

For the analysis, we performed a three-way repeated measures ANOVA using the grand average (summing across all trials) FAA as dependent variable. The first factor was trial type with two levels (normal and LOC). The second factor was electrode pair, also with two levels (F4 − F3 and FC2 − FC1). The third and last factor was alpha band (lower alpha = 8–10 Hz and higher alpha = 10–12). Effects only appearing in the data of individual subjects were not taken into account.

For the cluster analysis, we followed an established approach illustrated in Gramann et al. (2010) that used equivalent dipole modeling based on topographies of ICs across the whole subject sample (containing all correct trials for the normal and LOC conditions) to estimate the location of cortical sources underlying potential effects at the sensor level. This was performed in the following steps: first, ICs were computed on the individual EEG data (band pass filtered between 1 Hz and 100 Hz) using the CUDAICA implementation (Raimondo et al., 2012) of the Infomax ICA (Bell and Sejnowski, 1995). Next, dipole fitting was done using a simple three-shell spherical head model (registered to the Montreal Neurological Institute canonical template brain) implemented in the DIPFIT2-plugin (version 2.3) from the EEGLAB toolbox (Delorme et al., 2011; Oostenveld et al., 2011). Following this, the spectral activity of the resulting ICs was computed using the same data that was used for the scalp based analysis (approximately 15 min of EEG data that contained normal and LOC trials). To increase location accuracy of the final independent component clusters, all ICs that contained dipole coordinates with a residual variance greater than 15% have been automatically removed before clustering. As next step, IC clustering was performed across subjects and based on mean IC log spectra (between 3 Hz and 25 Hz), equivalent dipole locations as well as scalp maps as determining factors. The dipole locations were naturally three-dimensional and weighted by a factor of 20 to extract more closely spaced component clusters. All these measures were finally compacted into 10 dimensions via principal component analysis (Law and Jolliffe, 1987), since clustering algorithms may not work well with measures having more than 10 dimensions. Then, k-means clustering (Ding and He, 2004) was used with “k” set to 23 as suggested by the EEGLAB toolbox. Any outlier ICs were automatically removed if the distance to any cluster centroid in joint measure space was greater than three standard deviations from the mean. Afterward, the resulting IC clusters were inspected and ICs that exhibited activity that seemed to originate from non-brain artifacts were removed based on their activation spectra, scalp topographies and dipole location. Finally, anatomical regions were assigned to the centroids of the resulting component cluster using talairach coordinates and the Talairach web client (Lancaster et al., 2000).

## Results

### Scalp Based Analysis

**Figure 4:**
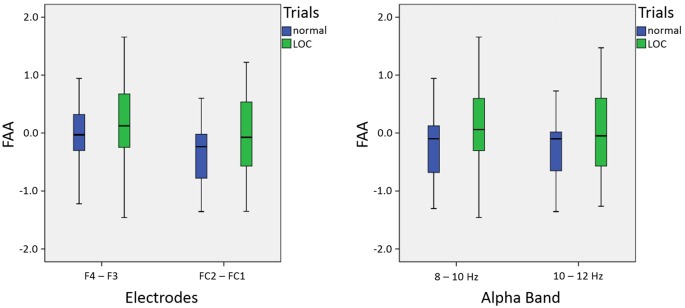
Box plots showing median values for electrodes (left; averaged across alpha bands) and alpha bands (right; averaged across electrodes): median values are indicated by black horizontal lines within the boxes. Top and bottom borders of the boxes represent the middle 50% of the data. Whiskers represent the smallest and largest values not classified as outliers or extreme values.

**Figure 5:**
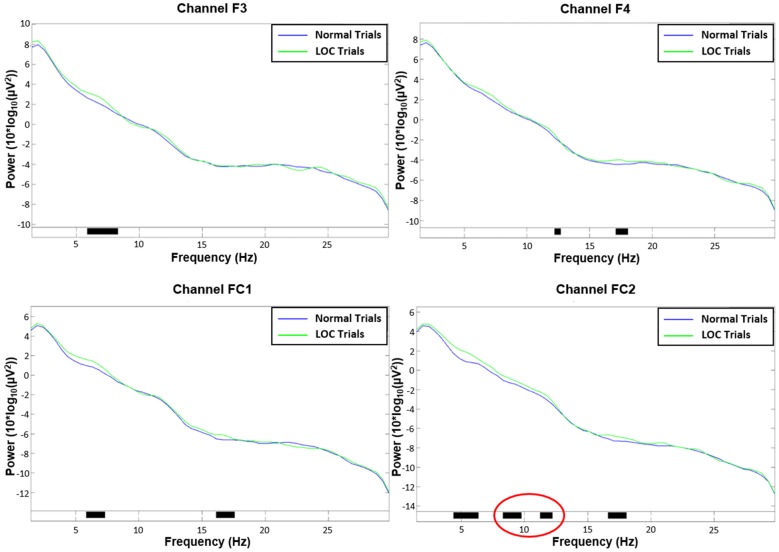
Channel spectra between 1 Hz and 30 Hz for all four channels used in the analysis. Top left: channel F3. Top right: channel F4. Bottom left: channel FC1. Bottom right: channel FC2. Significant differences (*p* < 0.05) are indicated with black bars. Please note that only channel FC2 shows significant activity in the alpha range (8–12 Hz; marked in red).

As expected, we found a main effect for trial type, *F*_(1,14)_ = 9.46, *p* < 0.01, *η*^2^ = 0.40. LOC trials showed stronger left frontal activation (due to higher right frontal alpha power) than normal trials. There was no significant main effect for electrode pair, *F*_(1,14)_ = 2.15, n.s. Furthermore, there was no significant main effect for alpha band, *F*_(1,14)_ = 0.65, n.s. Finally, none of the interactions exhibited significant effects (all *F*-values < 1.5). A topographic plot that illustrates the main effect for trial type can be seen in Figure 3 (right side), median values are shown in Figure 4 and channel spectra for all electrodes are shown in Figure 5.

### Cluster Analysis: Identifying Sources Underlying the FAAs Found at the Sensor Level

**Figure 6:**
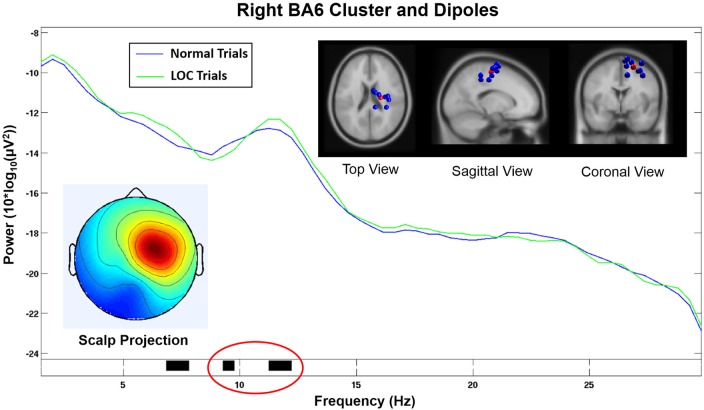
Component cluster in or near right Brodmann Area 6 (BA6). The spectrum shows a significant decrease (*p* < 0.05) in the lower alpha range (black horizontal bar marked in red) and a significant increase (*p* < 0.05) in the upper alpha range (black horizontal bar marked in green) during LOC. Top right shows the location of individual dipoles (blue) as well as the cluster centroid (red). Bottom left shows the corresponding scalp projection (red colors indicate higher cluster weights).

The cluster analysis yielded an IC cluster that contained data from nine subjects and showed spectral activity likely related to frontal laterality responses. The spectral activity of the component cluster projected strongly onto an area close to electrode FC2 (see Figure 6, bottom left). The scalp projection of this cluster showed the highest correlation (*r* = 0.64, *p* < 0.001, sig.; see Table 1) with the topographical difference plot (see Figure 3, right), when compared with the scalp projections of all other component clusters that exhibited a significant effect in the wide alpha band. Hence, the topographies show a unique, high similarity.

**Table 1:** Correlation coefficients between difference plot and all component clusters with significant activity in the alpha band.

Cluster	right BA39	left BA6	right BA6	right BA10	right BA37
ρ	**−0.24**	**0.17**	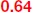	**0.38**	**−0.43**
All correlations significant at *p* < 0.001

*Please note that the right Brodmann Area 6 (BA6) cluster displays the highest correlation coefficient (marked in red), indicating the largest overlap between the component cluster and the difference plot*.

This component cluster was located in or near the right supplementary motor area (SMA) in Brodmann Area 6 (BA6; see Figure 6) and showed decreased lower alpha activity (*p* < 0.05) and increased higher alpha activity (*p* < 0.05) during LOC trials.

**Figure 7:**
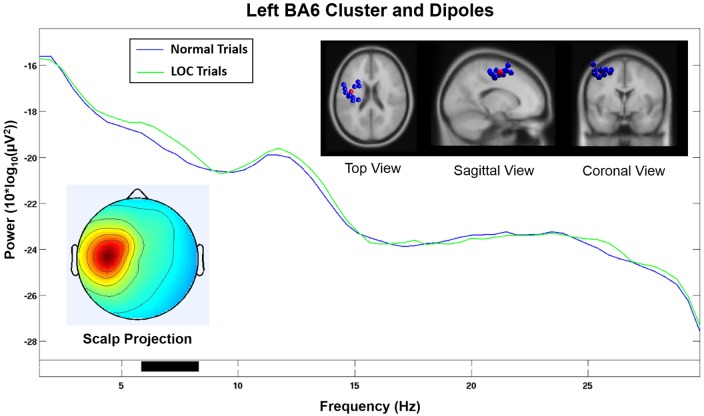
Component cluster in or near left BA6. Note that the spectrum does not show a significant increase (*p* < 0.05) in the alpha range during LOC. Top right shows the location of individual dipoles (blue) as well as the cluster centroid (red). Bottom left shows the corresponding scalp projection.

The homologous cluster located in or near the left BA6 (see Figure 7) and contained data from 11 subjects did not show significantly increased alpha power during LOC. A visual inspection of the activation spectra of both clusters did not show any indication of electromyographic contamination.

## Discussion

We could demonstrate that the complex user state in the focus of this article, namely the feeling of perceived LOC, displays some of the characteristic neural signatures of affective processes (i.e., brain laterality responses; Demaree et al., 2005). In particular, our scalp based analysis revealed a frontal asymmetry in the alpha band, indicating an increase in left frontal activity (due to an increase in right frontal alpha power) for incorrect stimulus rotation (LOC trials) as compared to normal trials with correct stimulus rotation. We further approximated the cortical sources that seem to contribute to the LOC-related FAA using equivalent dipole fitting of ICs, revealing opposing effects that could not be found at the electrode level.

### Frontal Alpha Asymmetry Indicates Affective Responses during Perceived Loss of Control

We found that trials with incorrect stimulus rotation (LOC) were accompanied with increased left frontal activity (due to increased right frontal alpha power). Such responses are commonly related to increased dominance, emotions associated with approach behaviors, as well as positive valence. While a shift toward positive valence would seem quite puzzling, an emotional shift toward more dominance and approach behaviors could be well explained with feelings of anger, hostility and contempt (Demaree et al., 2005), which possibly were experienced by subjects during LOC trials. These approach related responses would reflect engagement (Harmon-Jones et al., 2010), indicating that subjects made efforts to regain control of the system. This interpretation appears most likely, especially since no behavioral alternative was available (subjects could only press one button), previously formed strategies to solve the task did not work anymore due to the violation of task rules and the random nature of the occurring LOC trials made it difficult to adapt those strategies. Interestingly, the fact that the system did not respond properly anymore, impeding task performance, did not seem to result in task disengagement, since task disengagement should have been reflected in decreased left frontal activation, thereby implying avoidance behaviors resulting in feelings like frustration.

### Component Cluster Analysis Indicates Differential Effects Not Visible at the Sensor Level

Scalp electrodes record a mixed sum of cortical activity which can distort results and lead to incorrect conclusions. ICA uses a linear transformation of the scalp signal to separate the recorded activity into different data sources. In combination with equivalent dipole fitting it is possible to investigate the cortical dynamics that underlie the signals recorded at the sensor level. We found a component cluster that was located in or near BA6 displaying a scalp projection (see Figure 6, bottom left) closely resembling the laterality effect found in the alpha power difference plots (see Figure 3, right). This cluster exhibited an alpha band activity during LOC trials that was decreased in the lower alpha band (*p* < 0.05) and increased in the upper alpha band (*p* < 0.05; see Figure 6). Similar results have been found in a study by Reuderink et al. (2013). The authors found that activity in the lower alpha band was related to the valence dimension, while activity in the upper alpha band was related to the dominance dimension. However, our investigation did not exhibit this effect at the electrode level which is the basis of most other FAA related analyses. The reason for this discrepancy is probably due to the fact that scalp electrodes record a mixture of multiple different cortical sources, which can distort effects based on single cortical sources. However, the sample was too small to put a strong emphasis on the results of the cluster analysis and further studies are needed to support these findings. Nevertheless, we see this as further evidence that that the FAA effect might be more complex than previously assumed. A separate analysis of different alpha bands in combination with source based analyses might allow to disentangle different responses that are usually associated with this measure.

The component cluster that we found to be related to the FAA induced by LOC was located in or near BA6, which plays a major role in the planning of motor responses. In line with this function of BA6, previous research has indicated that emotional experiences are always associated with certain motor response tendencies (e.g., Önal-Hartmann et al., 2012), for instance, we reach for positive stimuli or push away negative ones. BA6 is composed of the premotor cortex and, medially, the SMA. The right BA6 cluster centroid was located closest to the SMA (see Figure 6, top right). The SMA has been shown to be active during motor action under affective influence. For example, SMA activation was found for emotional conflict in a study by Ochsner et al. (2009) in an affective flanker task. Another study by Oliveri et al. (2003) found enhanced motor evoked potentials in the SMA when using emotional stimuli. The authors conclude that the SMA plays a role when emotional experiences are transformed into motor actions. This evidence is in line with our expectations concerning the emotional effects of the paradigm used in our study.

Our findings are further supported by the fact that the homologous component cluster located on the left hemisphere and projected onto electrodes F3 and FC1 (see Figure 7) did not show a significant increase in the alpha band during LOC trials. Furthermore, the scalp projection of this component cluster did not exhibit a strong similarity (*r* = 0.17, *p* < 0.001, sig.; see Table 1) with the topographical difference plot (see Figure 3, right). We therefore conclude that the right BA6 component cluster is the main contributor to the laterality effect (measured via FAA) we found at the sensor level.

### Limitations and Outlook

Although we were able to identify affective responses in the LOC task as expected, this work has two main limitations. First, no classification has been conducted on the discovered laterality response. Future studies would need to adapt the LOC paradigm to make it suitable for a real-time classification approach. Such a paradigm should use motor imagery instead of button presses as input and implement a passive BCI for the automatic detection of affective processes that might interfere with the primary interaction mode. However, it is of utmost importance to state that building such a system is not trivial, since both the classification of motor imagery as control input as well as the detection of affective responses will not be 100% reliable and a separation of both influences on the scalp recorded EEG might prove difficult. Using ICs as classifier features might turn out to be a viable approach, but up to now it is quite unclear whether IC cluster remain stable when using them in such a dynamic system. Additionally, we also used healthy individuals as subject sample and it is yet unclear how our findings transfer to a clinical population.

Second, future studies should try to obtain more online measures of the subjective user states during interaction with the system. Since classification is done on a trial by trial basis, non-intrusive approaches would be ideal (e.g., heart rate or electrodermal activity). However, such approaches pose their own difficulties and usually do not work perfectly (Mauss and Robinson, 2009). Due to the necessary temporal density of user state measures, subjective ratings that can only be acquired in retrospect do also not seem very suitable for this paradigm. Nevertheless, they might provide an approximation of the general experience during the experiment and it is strongly recommended that future studies should implement them (e.g., affective ratings for all three dimensions from the pleasure-arousal-dominance model using the self-assessment manikin; Bradley and Lang, 1994). With such measures it might become possible to uncover inter-individual differences, thereby enabling a more differentiated perspective. For instance, we assume, that even if most subjects were not discouraged by the erratic behavior the system exhibited, some subjects might have been.

Furthermore, the current paradigm does not manipulate cognitive and affective processes separately and therefore these processes are confounded to a certain degree. Future studies should use paradigms that allow for such a manipulation to further disentangle the specific brain responses related to cognitive as well as affective responses. Potential candidates are the affective flanker task (Alguacil et al., 2013) or the affective n-back task (Passarotti et al., 2011).

Additionally, the findings from the cluster analysis should be seen as exploratory due to the small amount of channels and the rather small sample size. Furthermore, the use of the spherical head model can also introduce errors in the location estimate of the cortical sources. Moreover, template brain models were used to infer the location of the cortical sources which can introduce additional errors. Future studies should use high-density EEG recordings and individual brain scans to allow for more sophisticated source localization procedures. However, ICA-based analyses are of a more qualitative nature and therefore the resulting findings should be further substantiated using other approaches like combined EEG-FMRI recordings.

### Conclusion

Using FAA in combination with source localization approaches, we described brain responses in the EEG during naturalistic human-machine interaction that appear to be related to affective components of a complex user state (LOC). Our analyses helped to distinguish these responses from those related to the primary interaction mode (button press).

While this study should only be seen as a first step toward the investigation of the underlying factors that contribute to non-stationarities found at the sensor level, it still illustrates that complex user states involve multiple factors. Such factors might be used as additional context information in future BCI systems by extracting a multi-facetted user state model from the ongoing EEG. Such a user state model might help to increase reliability in uncontrolled settings by providing information which is usually hard to access.

## Author Contributions

SG is the main author, responsible for all tasks related to the publication. TOZ provided the data and was involved in the planning and writing of the article. JF was involved in the data analysis as well as writing of the manuscript. JB was helping with the data analysis. AK helped with the manuscript. KG was involved with the data analysis as well as writing of the manuscript. PG is the main coordinator also involved in all related tasks.

## Conflict of Interest Statement

The authors declare that the research was conducted in the absence of any commercial or financial relationships that could be construed as a potential conflict of interest.
